# An MRI Atlas of the Human Fetal Brain: Reference and Segmentation Tools for Fetal Brain MRI Analysis

**Published:** 2025-08-20

**Authors:** Mahdi Bagheri, Clemente Velasco-Annis, Jian Wang, Razieh Faghihpirayesh, Shadab Khan, Camilo Calixto, Camilo Jaimes, Lana Vasung, Abdelhakim Ouaalam, Onur Afacan, Simon K. Warfield, Caitlin K. Rollins, Ali Gholipour

**Affiliations:** 1 University of California Irvine, Department of Radiological Sciences, Irvine, CA 92617, USA; 2 Boston Children’s Hospital and Harvard Medical School, Department of Radiology, Boston, MA 02115, USA; 3 Northeastern University, Department of Electrical and Computer Engineering, Boston, MA 02115, USA; 4 ADIA Lab., Abu Dhabi, United Arab Emirates; 5 Department of Radiology, Massachusetts General Hospital and Harvard Medical School, Boston, MA 02114, USA; 6 Boston Children’s Hospital and Harvard Medical School, Department of Pediatrics, Boston, MA 02115, USA; 7 Boston Children’s Hospital and Harvard Medical School, Department of Neurology, Boston, MA 02115, USA

## Abstract

Accurate characterization of in-utero brain development is essential for understanding typical and atypical neurodevelopment. Building upon previous efforts to construct spatiotemporal fetal brain MRI atlases, we present the CRL-2025 fetal brain atlas, which is a spatiotemporal (4D) atlas of the developing fetal brain between 21 and 37 gestational weeks. This atlas is constructed from carefully processed MRI scans of 160 fetuses with typically-developing brains using a diffeomorphic deformable registration framework integrated with kernel regression on age. CRL-2025 uniquely includes detailed tissue segmentations, transient white matter compartments, and parcellation into 126 anatomical regions. This atlas offers significantly enhanced anatomical details over the CRL-2017 atlas, and is released along with the CRL diffusion MRI atlas with its newly created tissue segmentation and labels as well as deep learning-based multiclass segmentation models for fine-grained fetal brain MRI segmentation. The CRL-2025 atlas and its associated tools provide a robust and scalable platform for fetal brain MRI segmentation, groupwise analysis, and early neurodevelopmental research, and these materials are publicly released to support the broader research community.

## Background and Summary

Human cognition and performance are largely rooted in early brain development, a critical and vulnerable stage of maturation^1–8^. During this time, the brain undergoes a dynamic series of neurodevelopmental processes, including neuronal proliferation, neuronal migration, axonal development, synaptogenesis and apoptosis, the onset of myelination, and the formation of transient developmental zones within the telencephalic wall^9–18^. These zones eventually contribute to the formation of mature cerebral hemispheres. As a result, accurately characterizing early brain development in utero is essential to understand typical neurodevelopment and identify deviations from normal development. Fetal brain MRI, including quantitative analysis enables the study of these processes *in vivo*, but requires the incorporation of accurate brain atlases as templates for spatial normalization and as references defining normative spatiotemporal developmental trajectories. Consequently, significant efforts have been made to build early brain development atlases^19–26^ using magnetic resonance imaging (MRI), a modality that provides unmatched images of brain anatomy throughout the human lifespan including the fetal period^27^.

Constructing atlases from fetal MRI presents distinct challenges compared to adult atlas generation, primarily due to two factors. First, there are technical challenges in scanning fetuses with MRI. MRI is very sensitive to motion, and fetuses move near continuously during MRI acquisitions. Intermittent fetal and maternal respiratory movements prohibit precise spatiotemporal encoding that is necessary for real, high-resolution 3D MRI of the fetal brain anatomy. This challenge is exacerbated by the low level of MRI signal that is received from MRI body coils from the small fetal anatomy. Despite these challenges, the landscape of fetal MRI has changed dramatically in the past decade by the significant advances in retrospective slice-to-volume reconstruction (SVR) techniques^28–39^ and the subsequent development of fetal brain MRI atlases and processing tools^19–26^. Second, the brain structure and function change rapidly and dramatically during the fetal period of development. As a result, single static or 3D atlases are insufficient to capture these dynamic processes. Consequently, atlases covering this period should be spatiotemporal (i.e., dynamic or 4D) to accurately reflect the continuous and age-dependent nature of fetal brain development.

The construction of digital spatiotemporal MRI atlases of early brain development is relatively new: Kuklisova-Murgasova et al.^40^ developed a 4D probabilistic atlas of early brain growth from *in-vivo* MRI of 142 preterm infants in the 29 to 44 weeks post-menstrual age. They used pairwise affine registration of anatomy with kernel regression in age for atlas construction. Serag et al.^20^ used a non-rigid registration approach based on Bspline free-form deformations (FFD)^41^ and showed a marked improvement over the use of affine registration in atlas construction. Makropoulos et al.^42^ used a similar approach to construct a probabilistic spatiotemporal atlas of the neonatal brain from 420 segmented MRIs of neonates (including preterm neonates) scanned between 27 to 45 weeks post-menstrual age. To improve the FFD-based 4D atlas construction framework, Schuh et al.^43^ developed diffeomorphic registration based on the Log-Euclidean mean of inverse consistent FFD transformations.

For the fetal period of brain development, a recent review^44^ found 18 atlases presented in the literature, of which 12 are publicly available. Most of these atlases characterize the anatomy (structure) of the fetal brain based on 3D-reconstructed T2-weighted (T2w) MRI, but a few atlases also characterize the microstructure of the fetal brain based on diffusion-weighted MRI (dMRI) or the cortical surface based on cortical surface meshes reconstructed from anatomical MRI.

The first fetal MRI atlas was developed by Habas et al.^19^, based on 20 normal-appearing fetal brains scanned between 21–24 gestational weeks (GAs), offering age-specific T2w MRI templates and tissue probability maps of key brain structures such as cortical gray matter (cGM), white matter (WM), germinal matrix, and lateral ventricles (LV), using manual segmentation followed by groupwise registration and polynomial modeling of structural changes. Serag et al.^20^ used non-rigid FFD-based registration along with adaptive kernel regression on age to build a spatiotemporal atlas of fetal brain anatomy in a much broader age range of 23 to 37 gestational weeks. This atlas was built by using 3D-reconstructed^32^ T2w brain MRI images of 80 typically-developing fetuses. Dittrich et al.^21^ created an atlas from 32 fetuses between 20–30 GA using a semi-supervised method to reduce manual annotation by segmenting from partially labeled data. Gholipour et al.^22^ built a spatiotemporal atlas of the fetal brain anatomy from super-resolution reconstructed^30^ fetal brain MRIs of 81 healthy fetuses, covering 21–38 GA. This atlas, referred to as the CRL (Computational Radiology Lab) Fetal Brain Atlas, was built using kernel regression on age integrated into an ANTS^45^ group-wise symmetric diffeomorphic deformable registration framework. The CRL atlas uniquely offers detailed labels and tissue segmentations, and has been used as the standard space in several fetal SVR tools such as NiftyMIC^36^, MIALSRTK^35^, and NeSVoR^39^, and was used as a reference for comparison to the fetal brain ultrasound atlas developed by Namburete et al.^46^.

Urru et al.^47^ aligned the CRL fetal atlases with neonatal atlases from Serag et al.^20^, to build a unified fetal and neonatal label representation to support perinatal brain segmentation and analyses. In other atlas construction efforts, Li et al.^48^ constructed a fetal brain atlas from 35 Chinese subjects between 23–36 GAs at 2-week intervals, using deformable registration without explicit region of interest (ROI) definitions. Wu et al.^25^ enhanced this by generating weekly templates from 89 Chinese fetuses between 21–35 GAs using MIRTK registration and label propagation from the CRL atlas, making it the first publicly available atlas focused on this population. Xu & Sun et al.^39^ built upon these efforts by using 90 high-resolution fetal MRIs acquired on a 3T scanner from 23–38 GAs to construct an atlas with 85 brain structures, including the hippocampus and amygdala, segmented using the Draw-EM tool. Fidon et al.^49^ presented the first fetal atlas targeting a clinical population—Spina Bifida Aperta (SBA)—from 37 fetuses between 21–34 GAs, using anatomical landmarks, nonlinear registration, and post-processed segmentations to create templates with labels for structures such as the corpus callosum, cerebellum, brainstem, and extra-axial CSF, supporting surgical planning and training of disease-specific deep learning models.

Khan et al.^24^ developed the first spatiotemporal atlas of the fetal brain microstructure based on fetal dMRI. This atlas was constructed from motion-robust reconstructed diffusion tensor images (DTI)^50^ of 67 fetuses, covered the GA range of 22 to 38 weeks, and was released and used along with the CRL T2w atlas to characterize fetal brain maturation in several studies, e.g.^26,51,52^. Notably, based on this atlas and the fetal DTI data, Calixto et al.^26^ have recently developed a spatiotemporal atlas of 60 distinct white matter tracts, including commissural, projection, and association fibers of the fetal brain between 23 and 36 weeks of GA. In other works, Chen et al.^53^ developed a DTI atlas of the fetal brain from dMRI scans of 89 fetuses from the Chinese population scanned in the GA range of 24 to 38 weeks.

Uus et al.^54^ have built a high-quality, high-resolution multi-modal spatiotemporal MRI atlas of the fetal brain in the GA range of 21 to 36 weeks from reconstructed T2w, T1-weighted (T1w), and dMRI scans of 187 fetuses from the developing Human Connectome Project (dHCP)^55^. This atlas, referred to as the dHCP fetal MRI atlas, contains T1w, T2w, and dMRI modalities, including fractional anisotropy, mean diffusivity, and orientation distribution function images of the fetal brain, as well as tissue segmentations including the developing white matter, cortical gray matter, cerebrospinal fluid, and major deep gray matter structures, with a total of 17 labels.

Our goal in this work was to leverage the most recent technical advances in fetal MRI processing along with improved fetal imaging on 3T MRI scanners to generate a spatiotemporal MRI atlas of the fetal brain with much higher quality than the 2017 CRL atlas, but also with unique and detailed segmentations of the fetal brain anatomy, including tissue segmentations with the transient compartments of the developing white matter (36 total structures), as well as anatomical parcellation (126 total structures) that do not currently exist in any other fetal MRI atlas. These detailed segmentations and labels are crucial to conduct studies on regional brain maturation, connectivity analysis, regional groupwise analysis, and gene expression analysis. In this paper, we present the CRL-2025 fetal brain atlas, its construction framework, atlas labels, and, importantly, an open source framework based on state-of-the-art registration, parcellation, and segmentation methods to support automatic segmentation and population-level neurodevelopmental analysis. The CRL-2025 spatiotemporal fetal brain atlas provides a high-resolution anatomical reference from 21 to 37 weeks of GA based on carefully processed scans of 160 fetuses, and is released along with the CRL dMRI atlas^24^ as well as tissue segmentations and parcellations on the dMRI atlas, which are also unique and new in the field. The dMRI atlas provides diffusion tensor maps and diffusion measures such as fractional anisotropy and mean diffusivity, as well as detailed delineations of tissue segmentations and parcellations. Together or separately, these atlases can be used in a wide range of applications such as spatial normalization, automatic segmentation through label propagation, regional morphometric and volumetric analysis, multi-modal analysis, tract segmentation, tractography, and connectivity analysis to study developmental trajectories at the population level. To enable rapid and accurate region-level analysis on both T2w and dMRI scans, we have developed and released efficient segmentation and parcellation pipelines based on 1) multi-atlas segmentation, and 2) deep learning, two distinct approaches that balance anatomical fidelity with computational scalability. To this end, we have trained and compared several deep neural networks, including nnUNet^56^, UNetR^57^, Swin U-NetR^58,59^ and an attention-guided lightweight MAMBA network^60^ (called EMM-Seg), to segment individual fetal brains. These tools and atlases are released to support the research community in this burgeoning area of research.

## Methods

### Fetal MRI data

Fetal MRI data used in this study were acquired on various MRI scanners through research studies conducted at Boston Children’s Hospital between 2014 and 2023. All studies and the imaging protocols were reviewed and approved by the Institutional Review Board, and written informed consent was obtained from all participants. Inclusion criteria for those studies were for pregnant women between the ages of 18 and 45 who volunteered for research fetal MRIs and did not have any contraindication for MRI. Exclusion criteria for this study included anyone with a contraindication for MRI, multiple gestation pregnancies, or fetuses with any known, suspected, or documented morphological brain abnormality or diagnosis. Scans that were eventually included in the atlas construction were selected from a large database based on both acquisition and reconstruction quality, prioritizing high signal-to-noise ratio and quality of the reconstructed fetal brain images. Following these criteria, a total of 194 fetal MRIs obtained from 160 fetuses were used for spatiotemporal atlas construction. Individuals were scanned up to three times, generally at least six weeks apart. For the majority (129) of the 160 participants only a single scan was used for atlas construction, while 28 participants contributed images from two separate visits, and 3 participants contributed images from three visits.

Fetal MRIs were performed on Siemens 3T MRI scanners (Siemens Healthineers, Erlangen, Germany): Trio (n=5), Skyra (n=134), Prisma (n=53), or Vida (n=1), and a single scan on a Philips 1.5T scanner. Generally, 18-channel body matrix coils were used until August 2017, after which a 30-channel body matrix coil was used. Full research fetal MRI sessions took up to 60 minutes (with up to 45 minutes of imaging) during which several image sequences were taken based on project needs at the time, with 10–20 minutes of the session dedicated to T2-weighted Half-Fourier Acquisition Single shot Turbo Spin Echo (HASTE) imaging, about 10–15 minutes to diffusion MRI (dMRI), and the rest to other sequences. Typical imaging parameters for HASTE sequences were: echo time 115–120 ms, repetition time 1400–1600 ms, flip angle between 120 and 160, slice thickness = 2–3 mm, in-plane resolution = 1 mm, which was achieved by using a variable matrix size (256×256−320×320) that was adjusted based on a variable field-of-view (256–320 *mm*^2^) to cover the anatomy (mother and fetus). The dMRI scans used in atlas construction comprised of 2–8 scans each along one of the orthogonal planes with respect to the fetal head. In each scan, 1 or 2 b=0s/mm2 images, and 12 diffusion-sensitized images at b=500s/mm2 were acquired. Acquisition parameters were: minimal TR (typically 3000–4000ms), TE=60ms, in-plane resolution=2mm, slice thickness=2–4mm.

**Figure 1.**
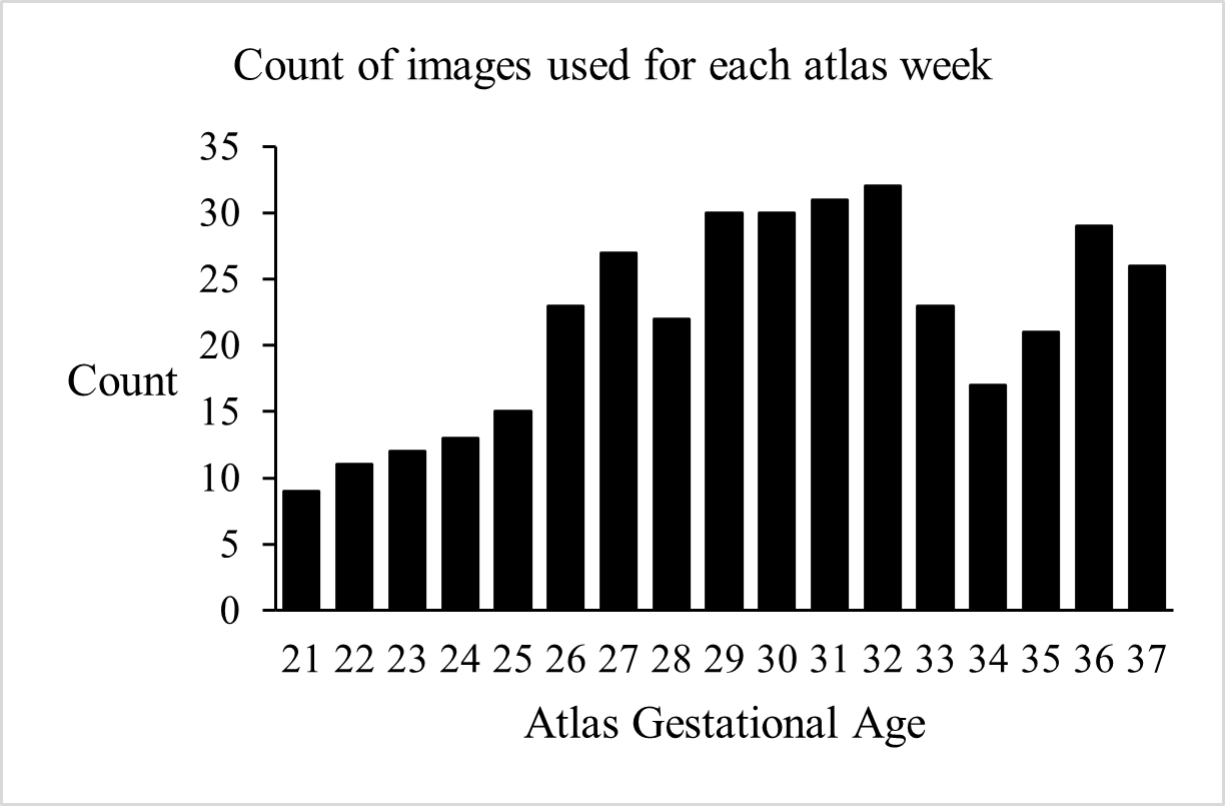
Frequency distribution of subjects contributed to atlas construction at each gestational age point in weeks.

**Figure 2.**
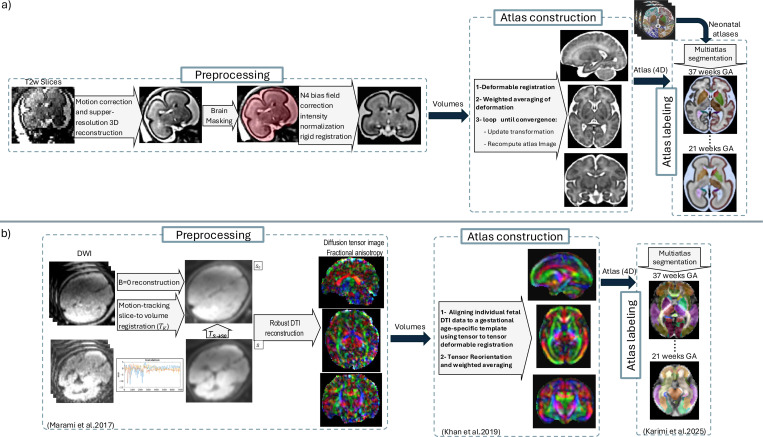
Overview of fetal MRI processing for spatiotemporal atlas generation a) Spatiotemporal anatomical atlas generation process based on fast T2-weighted MRI scans; b) Spatiotemporal diffusion MRI atlas generation process based on 1) motion-tracking based slice-to-volume registration for robust diffusion tensor image reconstruction^50^, 2) diffusion tensor atlas construction^24^, and 3) diffusion tensor atlas labeling^26^. Data, atlas construction, atlas labels, labeling procedures, automatic segmentation methods, and validations are discussed in this article.

A cutoff window of one gestational week was used to select subjects that contributed to atlas construction at every gestational week; so Fig.1 shows the histogram of the gestational age of the subjects used for atlas construction at every atlas week. Fig.2 shows an overview of the atlas construction process, which is discussed in the sections that follow. It should be noted that while we have built and released atlases at every gestational week, with the presented atlas construction framework, atlases can be built at any continuous age point (i.e., at any fractions of weeks or days).

### Pre-processing of T2w Images

Pre-processing of structural T2-weighted HASTE scans, illustrated in the top row of Fig.2, included the following steps: 1. Individual stacks were excluded if severe fetal or maternal motion persisted throughout the stack or image artifacts obscured the fetal brain; 2. Super-resolution reconstruction was performed using either the SVRTK^37^ or NiftyMIC^36^ toolkits; 3. B0-field inhomogeneity correction with the N4 algorithm^61^ and intensity normalization; 4. Intracranial cavity segmentation of the reconstructed brain image (”brain masking”); 5. Rigid registration to atlas anatomical space. Steps 3–4 were performed with in-house tools when SVRTK reconstruction was used, whereas NiftyMIC includes these steps in its reconstruction pipeline. Rigid registration to atlas space for SVRTK-processed reconstructions was performed with FLIRT^62^. Default settings were used for SVRTK and NiftyMIC was run with the alpha parameter set to 0.04. Prior to SVRTK reconstruction, a rough ellipsoid mask was drawn over the fetal brain in one reference stack in ITK-SNAP^63^. Prior to NiftyMIC reconstruction, the NiftyMIC fetal brain extraction pipeline was used to crop the input images. Each reconstruction included three to eighteen stacks (mean=8 stacks), with at least one axial, one coronal, and one sagittal stacks without significant motion artifacts.

### Spatiotemporal anatomical atlas construction

For spatiotemporal atlas construction, we used a modified recursive mean intensity function to incorporate an estimate of the longitudinal deformations of the atlas space across time points, as presented by Schuh et.al.^23^.

To construct the spatiotemporal atlas, we defined the mean intensity and anatomical shape at iteration k as:

(1) $${\bar{I}}_{k}(t)=\sum_{i=1}^{n}w_{i}(t)\left({\tilde{I}}_{i}\circ T_{k,i}^{-1}(t)\right)$$

Here, ${\tilde{I}}_{i}(x)$ refers to the globally normalized image for subject i, and $T_{k,i}$ denotes the estimated diffeomorphic mapping from the subject’s native space to the common atlas space at time t, during iteration k.

The temporal regression weights $w_{i}(t)$ are derived from normalized Gaussian functions centered at age t, with variance $\sigma_{t}^{2}$ adapting to the time point. Specifically: $\sigma_{t}$, i.e., $w_{i}(t)=\frac{g_{i}(t)}{\sum_{j=1}^{n}g_{j}(t)}$ where $g_{i}(t)=\frac{1}{\sigma_{t}\sqrt{2\pi }}\exp\left(\frac{-{\left(t_{i}-t\right)}^{2}}{2\sigma_{t}^{2}}\right)$. To restrict the spatial influence of far-off time points, all weights outside of a kernel width of one week were truncated. The mapping $T_{k,i}(t)$ is composed of a residual deformation ${\bar{\phi }}_{k}(t)$ and a subject-specific transformation $\phi_{k,i}$:

(2) $$T_{k,i}(t)={\bar{\phi }}_{k}(t)\circ \phi_{k,i}$$

where

(3) $${\bar{\phi }}_{k}(t)=\exp\left(-\sum_{i=1}^{n}w_{i}(t)\log\phi_{k,i}\right)$$

denotes the weighted Log-Euclidean mean^64^ of the inverse subject-to-atlas deformations, $\phi_{k,i}$, obtained via stationary velocity free-form deformation (SVFFD) registration of ${\tilde{I}}_{i}$, presented in^23^, to the age-matched average image from the previous iteration, i.e., ${\bar{I}}_{k-1}\left(t_{i}\right)$. The logarithmic maps $\text{log}\;\phi_{k,i}$ correspond directly to the stationary velocity fields (SVFs) computed during registration. The cross-sectional diffeomorphism mapping ${\tilde{I}}_{i}$ into the atlas space at $t_{i}$ is given by

(4) $$\phi_{k,i}=\exp\left({\text{v}}_{k,i}\right)$$

where

(5) $${\text{v}}_{k,i}=\text{arg}\;\underset{\text{v}}{\text{min}}E\left({\tilde{I}}_{i},{\bar{I}}_{k-1}\left(t_{i}\right),\text{v}\right)$$

is the (local) minimum of the energy given presented by Schuh et.al.,^23^, and ${\text{v}}_{0}=0$.

To track the sequence of spatial deformations applied at each time point t, and to use these deformations to derive a longitudinal coordinate map that relates an observed age $t_{i}$ to any other time point, thereby correcting for anatomical mismatches in the co-domain, i.e.,

(6) $$\Psi_{k,i}(t)={\bar{\varphi }}_{k}(t)\circ \Psi_{k-1,i}(t)\circ {\bar{\varphi }}_{k}^{-1}\left(t_{i}\right)=\left(\prod_{s=1}^{k}{\bar{\varphi }}_{s}(t)\right){\left(\prod_{s=1}^{k}{\bar{\varphi }}_{s}\left(t_{i}\right)\right)}^{-1}$$

where, Π denotes a sequential composition of functions, and $\bar{\varphi }$ corresponds to the refined Log-Euclidean mean of the transformations. This formulation, which avoids recursion, is based on the assumption that the initial longitudinal deformation between any two time points—specifically from $t_{i}$ to t—is the identity map, i.e., $\Psi_{0,i}(t)=\text{Id}$. As each transformation is expressed via the exponential of a SVF, their compositions can be efficiently approximated using the Baker–Campbell–Hausdorff expansion. This ensures that the resulting transformations stay consistent within the SVFFD model.

The composition of the subject-to-atlas transformation given by Equation (4), obtained by registering the i-th image to the template ${\bar{I}}_{k-1}\left(t_{i}\right)$, with the longitudinal deformation defined in Equation (6), yields an age-specific deformation, i.e.,

(7) $$\varphi_{k,i}(t)=\Psi_{k-1,i}(t)\circ \phi_{k,i}$$

The residual atlas deformation is now given by the Log-Euclidean mean of the age-adjusted atlas-to-subject transformations. It is thus given by

(8) $${\bar{\varphi }}_{k}(t)=\exp\left(-\sum_{i=1}^{n}w_{i}(t)\log\varphi_{k,i}(t)\right)$$

which are further used to redefine the total age-dependent deformations, i.e.,

(9) $$T_{k,i}(t)={\bar{\varphi }}_{k}(t)\circ \varphi_{k,i}(t)={\bar{\varphi }}_{k}(t)\circ \Psi_{k-1,i}(t)\circ \phi_{k,i}$$

Based on the presented equation, Algorithm 1 is used to construct the spatio-temporal atlas.

**Algorithm 1:** Spatio-temporal Atlas Construction

1:	**Input:** Globally normalised images ${\tilde{I}}_{i}$, previous transformations ${\text{T}}_{k-1,i}$, weights $w_{i}(t)$	
2:	**Output:** Updated atlas templates and transformations	
3:	**for all** $t_{i}$ such that $\left|t_{i}\right|\leq n$ **do**	
4:	Generate template images ${\bar{I}}_{k-1}\left(t_{i}\right)$ given ${\tilde{I}}_{i}$ and ${\text{T}}_{k-1,i}$	▷ Eq. (1)
5:	Compute $\phi_{k,i}=\exp\left({\text{v}}_{k,i}\right)$ from i-th image to template at $t_{i}$	▷ Eq. (4)
6:	**for all** $t\in \left\{t_{j}|w_{i}\left(t_{j}\right)>0\right\}$ **do**	
7:	Compose maps $\Psi_{k-1,i}$ given $\Psi_{k-2,i}$ and ${\bar{\varphi }}_{k-1}$	▷ Eq. (6)
8:	Compose maps $\phi_{k,i}$ with $\Psi_{k-1,i}$	▷ Eq. (7)
9:	**end for**	
10:	**end for**	
11:	**for all** observed $t_{i}$ **do**	
12:	Compute Log-Euclidean means ${\bar{\varphi }}_{k}$	▷ Eq. (9)
13:	**end for**	

### Atlas labeling and segmentation

The spatiotemporal fetal brain MRI atlas offers a high signal-to-noise representation of normal fetal brain anatomy across gestation, serving as a robust foundation for tissue-type segmentation and anatomical labeling. Segmentations for the T2-weighted CRL-2025 Atlas were generated in a manner similar to (and utilized) the pre-existing atlas and individual subject atlas images, as described and validated for the segmentation of individual subjects in Gholipour et al. 2017^22^ and Rollins et al. 2021^65^. In short, the Advanced Normalization Tools (ANTs)^45^ were first used to perform symmetric diffeomorphic registration of reference atlas images within one week gestational age to each CRL-2025 Atlas image, followed by automatic multi-atlas segmentation with the Probabilistic STAPLE algorithm^66^, producing two segmentations: a tissue segmentation delineated by structure (including cortical plate, the developing white matter, subcortical structures, cerebrospinal fluid spaces (CSF), cerebellum, brainstem, and more- a modified protocol sourced from the neonatal ALBERT atlas^67^, plus white matter compartments^65^) and a regional segmentation organized by location (gyri, sulci, lobe, etc.), corresponding to the atlas labels presented in Blesa et al. 2016^68^. For manual segmentations and parcellations we closely followed the protocol and process detailed in Gholipour et al.^22^. Manual segmentations and refinements, following this protocol, were done in multiple rounds by four experts each with several years of experience in fetal MRI segmentation under the supervision of a neurologist and a neuroanatomist both with decade-long experience in fetal neuroanatomy based on histology and MRI. Automatic segmentation labels propagated at each age from the CRL-2017 atlas to the CRL-2025 atlas were all checked and manually corrected in ITK-SNAP by an expert with ten years of experience in fetal MRI annotation. White matter compartment labels were only applied to atlas images up to gestational age 31 weeks, as these compartments became less visible on MRI after 31 weeks and disappeared with advanced gestational age^22,69^. Compared to the CRL-2017 atlas^22^ supplemented with white matter compartment labels^65,69^, the tissue segmentation protocol has been further modified for the CRL-2025 Atlas. Small, low-contrast, and under-utilized labels for hippocampal commissure and subthalamic nuclei have been removed and incorporated into surrounding labels; new labels were assigned to CSF spaces, cavum septum and the third and fourth ventricles; and a bilateral vermis label was added to the cerebellum. Each CRL-2025 Atlas segmentation required approximately one hour for automatic registration and multi-atlas segmentation and two to four hours of manual editing for updates and corrections. In total, the tissue segmentation includes 36 labels and the region segmentation includes 126 labels. Tissue and region atlas segmentations can be crossed to produce cortical parcellations.

### Fetal diffusion MRI atlas and labels

The fetal diffusion MRI atlas was developed by Khan et al.^24^, which provides a population-averaged diffusion tensor imaging (DTI) atlas of the fetal brain across 22 to 38 gestational weeks. They first reconstructed individual fetal diffusion tensor images using motion-corrected slice-to-volume registration. Each volume was then warped using a two-step registration process that first aligns individual fetal DTI data to a gestational age-specific template using affine and non-linear transformations, followed by tensor reorientation using finite strain methods to ensure anatomical and directional consistency. The registered tensors were then averaged in the log-Euclidean space to produce a spatiotemporally consistent atlas that preserves diffusion anisotropy and principal diffusion directions. This approach enabled, for the first time, accurate mapping of developmental trajectories in white matter microstructure at a fine spatiotemporal resolution based on in-vivo MRI of healthy fetuses through mid to late gestation^24^. A significant number of major tracts were identified this atlas (See Figure 7 in Khan et al.^24^).

Tissue segmentations and cortical parcellations were initiated on the DTI atlas through diffeomorphic deformable registration of the CRL age-equivalent T2w atlas to the corresponding mean diffusivity map of the DTI atlas. Subsequently, using the computed deformation fields, labels from age-matched fetal brain atlases were mapped to each DTI atlas. The propagated labels for major tissue classes and anatomically defined cortical and subcortical structures were then carefully reviewed and manually refined by an expert with more than five years of experience in fetal neuroanatomy on MRI and dMRI and checked by a neuroradiologist with more than ten years of experience in fetal MRI.^26^

### Automatic Segmentation

Many advanced downstream tasks in quantitative fetal brain MRI analysis rely on regional and/or tissue-level segmentations. The CRL-2025 atlas uniquely provides labels on both anatomical and diffusion MRI atlases that enable automatic segmentations. We have explored and developed two mainstream techniques for automatic fetal brain MRI segmentation based on these atlases: 1) multi-atlas segmentation, and 2) deep-learning based segmentation, detailed in the following subsections.

#### Multi-atlas segmentation

Multi-atlas segmentation (MAS) involves three main steps: 1) deformable registration of atlases/templates to the query subject anatomy, 2) applying the calculated deformations to propagate labels from the atlases/templates to the query subject space, and 3) label fusion to compute a consensus label for each voxel of the query image. Our MAS algorithm uses ANTs diffeomorphic deformable registration^45^ with hierarchical rigid, affine, and symmetric normalization by maximizing cross-correlation similarity metric for registration, interpolation for label propagation, and probabilistic STAPLE^66^ for label fusion. However, various other tools, such as MIRTK deformable registration^41,43,70^, other similarity metrics^71^, or other label fusion techniques, e.g.^72,73^ can be used. MAS segmentation of reconstructed T2-weighted or DTI fetal brain images should employ age-matched atlases/templates. When using the CRL-2025, for example, one shall use atlases within one week of GA from the query subject’s GA. It should be noted that if a larger number of individual-subject templates with reliable labels are available, they can be used as multiple atlases in MAS (separately or in combination with the CRL-2025 atlases). For more detailed description of MAS and its validation we refer to the literature cited above, and for an application and validation in fetal MRI we refer to Gholipour et al.^22^.

#### Deep learning segmentation

Deep learning based segmentation models have gained popularity due to their fast inference capabilities compared to conventional MAS methods, as well as their ability to learn direct mappings from images to segmentations without requiring image registration or label propagation. We developed, trained and evaluated several deep learning models for fetal brain MRI segmentation. Specifically, we trained and evaluated three state-of-the-art segmentation models, based on the nnU-Net^56^, UNETR^57^, and SwinUNETR^58^ architectures; additionally we designed a lightweight model, EMM-Seg, which integrates Residual Vision MAMBA (RVM) blocks into a U-Net backbone and employs depthwise separable convolutions (DWConvs)^60^ for improved efficiency. Inspired by LightM-UNet ^60^, EMM-Seg reduces parameters while preserving spatial details. This architecture features an encoder with RVM-enhanced convolutional layers, a bottleneck for global context, and a decoder with skip connections for accurate reconstruction. It balances efficiency and accuracy using a tailored loss function that optimizes both voxel-wise precision and shape fidelity. More details of this architecture and the implementation can be found in the released code repository. The encoder applies convolutional layers interleaved with RVM blocks to hierarchically extract multi-scale features while progressively increasing the receptive field. The bottleneck aggregates global contextual information, and the decoder reconstructs high-resolution segmentation maps using skip connections and lightweight upsampling. DWConv layers replace standard convolutions throughout, significantly reducing the number of parameters while preserving spatial structure.

A DWConv layer processes each input channel *c* independently as

(10) $$Y_{c}(i,j,k)=\sum_{u,v,w}X_{c}(i+u,j+v,k+w)\cdot K_{c}(u,v,w),$$

with parameter count

(11) $$P_{\text{sep}}=C_{\text{in}}k^{2}+C_{\text{in}}C_{\text{out}},$$

compared to $P_{\text{std}}=C_{\text{in}}C_{\text{out}}k^{2}$ for standard convolutions.

Each RVM block performs linear projection and self-similarity mixing (SSiM):

(12) $$F^{\prime }=\mathrm{Norm}(F)+W_{1}F+b_{1},$$

(13) $$S_{ij}=\mathrm{softmax}\left(\frac{F_{i}F_{j}^{T}}{d}\right),$$

(14) $$F_{\text{out}}=F^{\prime }+\mathrm{SSiM}\left(F^{\prime }\right),$$

where $F_{i}$ and $F_{j}$ are feature vectors at different spatial positions. This design models global context with linear complexity $O\left(d\cdot d_{s}\right)$ instead of the quadratic $O\left(d^{2}\right)$ cost of traditional self-attention.

The decoder reconstructs voxel-wise class probabilities $P\in {\mathbb{R}}^{H\times W\times D\times K}$ via

(15) $$P(i,j,k,c)=\frac{\exp\left(F_{\text{dec}}(i,j,k,c)\right)}{\sum_{c^{\prime }=1}^{K}\exp\left(F_{\text{dec}}\left(i,j,k,c^{\prime }\right)\right)}.$$

Skip connections add encoder features to upsampled decoder features:

(16) $$F_{\text{up}}(i,j,k)=\text{Interpolate}\left(F_{\text{low}}(i,j,k)\right)+F_{\text{skip}}(i,j,k).$$

The model is trained end-to-end with a hybrid loss that combines voxel-wise cross-entropy and Dice loss:

(17) $$L=L_{\text{CE}}+\varepsilon L_{\text{Dice}},$$

where ε balances voxel-level accuracy and shape consistency. This dual loss enforces robust feature representation while maintaining fine anatomical boundaries. By using DWConv, RVM blocks, and decoupling global context modeling from class-specific outputs, EMM-Seg achieves a parameter count that scales primarily with input size and feature dimensions rather than the number of classes K. This makes it more efficient than transformer-based models while maintaining accuracy.

Models were implemented in PyTorch. All experiments, including training and testing, were performed on a computer workstation with an NVIDIA RTX A5000 GPU for 200 epochs using the AdamW optimizer^74^ with a learning rate of 10^−4^ and a PolyLR scheduler with a batch size of one. We trained, tested, and compared four deep learning models (nnU-Net, UNETR, SwinUNETR, and EMM-Seg) on two fetal brain MRI datasets: an in-house dataset collected by our group at Boston Children’s Hospital (BCH), and the publicly available FeTA (Fetal Tissue Annotation) dataset^75^. The BCH dataset included 177 subjects with T2-weighted fetal MRI scans spanning 20–38 weeks of GA. Tissue segmentations and parcellations on these individual subject images were generated through manual refinement of multi-atlas segmentations following the manual segmentation protocols that were described in the Methods section based on^22^. The FeTA dataset consisted of 80 subjects with publicly available fetal MRI scans covering 20–37 weeks GA, with expert-annotated segmentations for 7 anatomical labels, including cortical gray matter (GM), deep gray matter (DGM), white matter (WM), brainstem (BS), cerebellum (CB), cerebrospinal fluid (CSF), and ventricles (VEN). To ensure subject independence, we applied a subject-wise split to both datasets, reserving 25% of subjects for testing, with the remaining scans used for training and validation. Segmentation performance was assessed using the Dice Similarity Coefficient (DSC) and Hausdorff Distance (HD).

### Data Records

The CRL-2025 spatiotemporal fetal brain MRI atlas, including the T2w atlases and the DTI atlases, that span from 21 weeks of gestation to 37 weeks of gestation, and their associated labels, including tissue segmentations, parcellations (regional), and transient white matter compartments (visible until 31 weeks of gestation) are publicly available and can be accessed through Harvard Dataverse: https://dataverse.harvard.edu/dataverse/CRL2025Atlas/, or using DOI links https://doi.org/10.7910/DVN/QOO75G (T2w) and https://doi.org/10.7910/DVN/XWKCIE (DTI). Each atlas includes a label key text file that can be used in a viewer such as ITKSNAP^63^ for better visualization of the segmentations.

### Technical Validation

#### The spatiotemporal fetal brain MRI atlas

**Figure 3.**
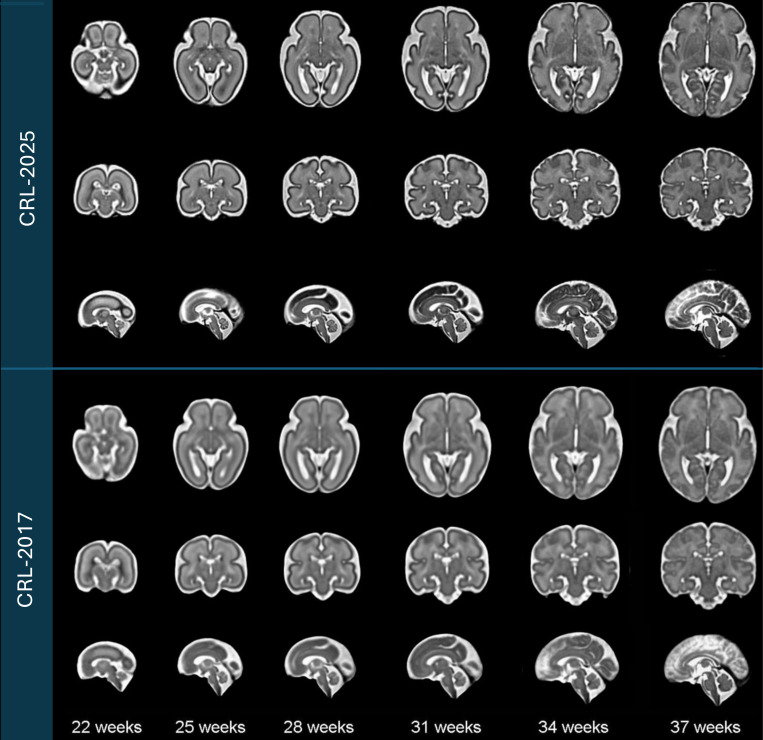
Comparison of spatiotemporal fetal brain MRI atlases ( CRL-2025 vs. CRL-2017) at six representative gestational ages: 22, 25, 28, 31, 34, and 37 weeks. Axial, coronal, and sagittal views are presented for each atlas at each age.

Fetal brain MRI scans were pre-processed following the procedures outlined in the Methods section. The processed images were then used to construct the spatiotemporal atlas according to Algorithm 1, which enables the generation of an unbiased average atlas at any continuous gestational age. Figure 3 displays axial, coronal, and sagittal views of the resulting atlas at representative gestational ages, alongside corresponding views from the CRL-2017 atlas for comparison^22^. The atlas shape and size complies with the older validated atlases, including the CRL-2017 atlas. Both visual inspection and quantitative analyses suggest that the CRL-2025 atlas much better preserves anatomical details and is sharper than the CRL-2017 atlas. For this, as shown in Table 1, we computed median edge sharpness to quantitatively assess the clarity of anatomical boundaries in each atlas. This metric, defined as the median gradient magnitude at anatomical edges detected within the atlas, reflects sharpness of typical tissue transitions. Across all gestational ages, CRL-2025 consistently exhibited higher median edge sharpness than CRL-2017, with differences ranging from +0.14 to +0.39. These results demonstrate that CRL-2025 provides more clearly defined anatomical boundaries throughout development, confirming its advantage for many applications such as atlas-based segmentation and analysis.

**Figure 4.**
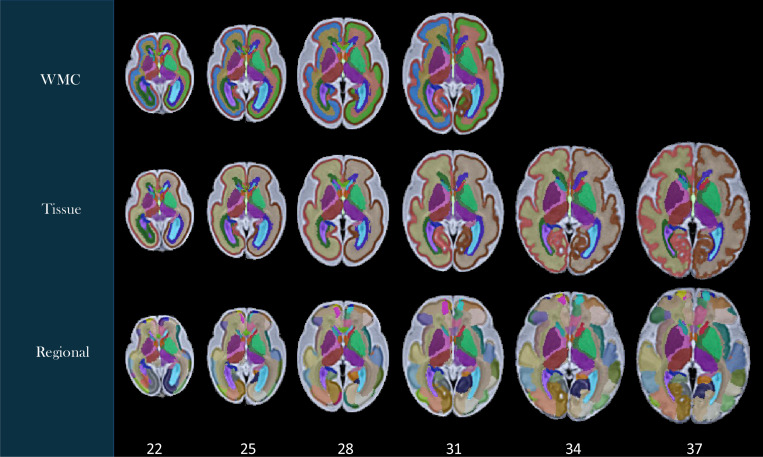
Tissue and regional segmentations and structural labels overlaid on axial views of the CRL-2025 spatiotemporal fetal brain MRI atlas at six representative gestational age (GA) weeks. All label schemes have subcortical structures including lentiform and caudate nuclei, internal capsules, thalami, and hippocampi separately on each hemisphere. Tissue segmentation labels (middle row) delineate the cortical plate-white matter boundary, CSF, and subcortical structures. In the top row, the white matter of the tissue segmentation is divided into white matter compartments (WMC) including the ventricular and intermediate zones and the subplate. These transient WMCs gradually disappear and were not clearly observable on the atlases beyond 31 weeks. The bottom row displays regional segmentations which are useful for regional and connectivity analyses.

**Figure 5.**
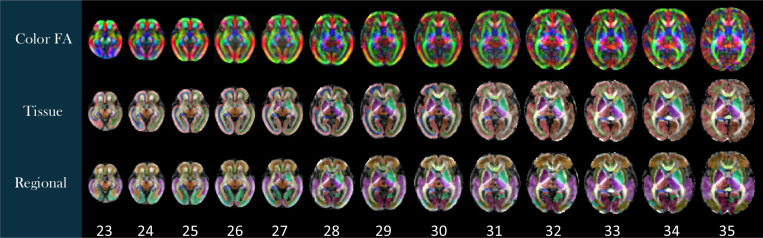
Diffusion Atlas, gestational age (GA) weeks 23–35. Top row: Color fractional anisotropy (FA). Middle row: Tissue segmentation. Bottom row: Regional segmentation.

Labels were generated on the spatiotemporal fetal brain MRI atlases following the procedure described in the Methods section. As illustrated in Figure 4, these labels include 1) the transient compartments of the developing white matter (WMC) for all atlases less than 32 weeks, 2) tissue types, and 3) regions. For details of these labels, we refer to the text files in the data repository. Figure 5 shows the fetal DTI atlas^24^ (color anisotropy in the first row) along with its tissue segmentations (second row) and regional parcellations (third row) that are released in this edition with the CRL-2025 atlas. All of the segmentations and labels on the T2w atlas were carefully checked, manually refined, and validated in multiple rounds by four experts under the supervision of a neurologist and a neuroanatomist each with more than a decade of experience in assessing fetal neuroanatomy on MRI. All the segmentations on the DTI atlas were carefully checked, manually corrected, and validated by two experts with several years of experience in fetal neuroanatomy on MRI and dMRI. To further assess and validate the use of the atlases and their labels, we used the atlases for multi-atlas segmentation of individual subject fetal brain MRIs, and used multi-atlas generated, manually-corrected labels on individual subject images^22^ to train and test deep learning based segmentation models (as discussed in the Methods section) and presented in the next section.

#### Automatic Segmentation

Table 2 summarizes automatic segmentation performance of the four trained deep learning models on the BCH and FeTA datasets. Overall, the results show that all methods performed well on BCH data, whereas their performance was generally weaker on the FeTA data. This could have several sources: first, the FeTA data intentionally include low-quality reconstructed images (images with motion artifacts and those reconstructed from thick-slice acquisitions) and also include images from severely abnormal anatomies^75–77^. Abnormalities and low-quality images pose significant challenges in automatic segmentation. Second, the FeTA data include some noisy labels^78^, which limit the best achievable accuracy. Among the deep neural networks we tested, nnU-Net showed the best performance on the BCH data, whereas EMM-Seg performed best on the FeTA data. So it seems that with its efficient modeling of long range and short range dependencies the EMM-Seg showed better robustness and generalization in the presence of abnormalities and noise in the FeTA data. It is noted that EMM-Seg attained comparable accuracy to nnUnet and SwinUNetR while using only 96.89 GFLOPs and 2.89 million parameters.

**Figure 6.**
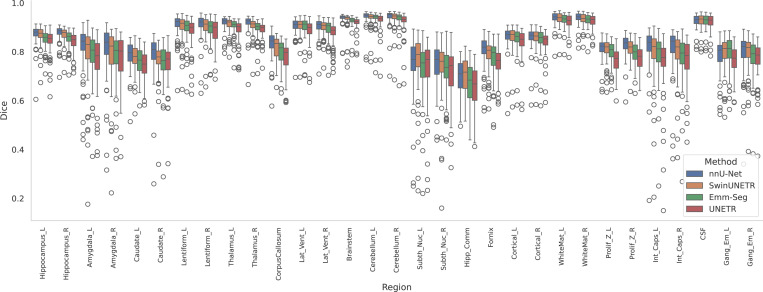
Boxplots of Dice performance metrics over the test sets for 31 distinct segmentation regions for the BCH dataset using four automatic segmentation models (EMM-Seg, UNETR, SwinUNETR, and nnUnet).

Fig. 6 presents boxplots of the DSC across the 31 segmentation regions for the BCH test subjects. The results indicate relatively reliable automatic segmentations were achieved by all models for most of the major structures such as the cortical plate, subplate, CSF, ventricles, corpus callosum, and thalamus. On the other hand, segmentations were more difficult, with lower DSCs and higher HDs, for small and/or challenging structures such as amygdala, caudate nuclei, subthalamic nuclei, hippocampal commissure, and fornix.

## Figures and Tables

**Table 1.** Median edge sharpness (gradient magnitude at anatomical boundaries) for CRL-2017 and CRL-2025 atlases across gestational ages.

GA	21	22	23	24	25	26	27	28

CRL-2017	1.008	0.938	0.986	0.950	0.911	0.884	0.886	0.883
CRL-2025	1.349	1.330	1.290	1.270	1.266	1.229	1.225	1.160

GA	29	30	31	32	33	34	35	36

CRL-2017	0.867	0.842	0.809	0.760	0.696	0.632	0.605	0.622
CRL-2025	1.159	1.177	1.093	1.037	0.963	0.848	0.799	0.764

**Table 2.** Comparison of the performance of four deep learning models for automatic fetal brain MRI tissue segmentation: average and standard deviation of Dice and HD values over test sets on BCH and FeTA datasets.

Methods	BCH (31 regions)		FeTA (19 regions)		GFLOPs	Params (M)

	Dice	HD	Dice	HD		
nnU-Net	**0.853±0.09**	**0.984±0.44**	0.757±0.161	3.304±2.877	479.73	31.18
UNETR	0.821±0.101	1.33±0.64	0.764±0.165	3.941±2.934	329.26	96.85
SwinUNETR	0.845±0.093	1.04±0.41	0.766±0.159	3.255±2.810	201.93	15.70
EMM-Seg	0.835±0.094	1.13±0.45	**0.773±0.150**	**3.131±2.792**	**96.89**	**2.89**

## Code Availability

The multi-atlas segmentation code can be found at https://github.com/IntelligentImaging/CRL-2025-MAS. The deep learning based segmentation codes can be found at https://github.com/IntelligentImaging/CRL-2025. These repositories include resources for customizing workflows for automatic segmentation model training and deployment, links to the trained models, and data analysis tools to reproduce figures presented in the manuscript.
